# Prevalence and determinants of comprehensive eye care in a group of patients with diabetes: a cross-sectional study in a sub-Saharan African setting

**DOI:** 10.1186/s13104-018-3265-1

**Published:** 2018-02-27

**Authors:** Ahmadou M. Jingi, Jean Jacques Noubiap, Yannick Bilong, Aurel T. Tankeu, Côme Ebana Mvogo

**Affiliations:** 1 Department of Internal Medicine and Specialties, Faculty of Medicine and Biomedical Sciences, Yaounde, Cameroon; 2 Department of Medicine, Groote Schuur Hospital and University of Cape Town, 7925 Observatory, Cape Town, South Africa; 3 Department of Ophthalmology, Faculty of Medicine and Biomedical Sciences, Yaounde, Cameroon

**Keywords:** Diabetes, Eye, Fundoscopy, Sub-Saharan Africa

## Abstract

**Objectives:**

We aimed to investigate the determinants of comprehensive eye examination in diabetes patients. We conducted a cross-sectional study at the eye department of the Douala General Hospital. Adult patients with diabetes were consecutively interviewed on the history of their diabetes. Main outcomes were a first ever comprehensive eye examination including fundoscopy, and diagnosis-to-fundoscopy time.

**Results:**

52 patients were included of whom 59.6% were males with a mean age of 55.9 ± 10.9 years. 51.9% have had counselling on the risk of visual impairment and blindness due to diabetes, and 61.5% [95% CI 47–74.7] have had a comprehensive eye examination. Of those with a first ever fundoscopy, only 21.9% had the test performed within 1 year of diagnosis. Thus, after an average of 10 years of the diagnosis of diabetes, 13.5% (7/52) of patients have had a comprehensive eye examination within 1 year of diagnosis. Only dose with duration of diabetes of more than 10 years were 7–24 times more likely to have a comprehensive eye examination. In summary, patients with diabetes in this low-income setting do not receive a comprehensive eye care as recommended. Most patients will get an eye examination at least 10 years after the diagnosis of diabetes.

**Electronic supplementary material:**

The online version of this article (10.1186/s13104-018-3265-1) contains supplementary material, which is available to authorized users.

## Introduction

Diabetes has reached epidemic proportions with the greatest burden on low-to-medium income settings [1], where it is under-diagnosed, under-investigated, and under-treated [2]. For, instance, it affects about 6.5% of adults Cameroonians [3]. This high disease burden is associated with low availability of investigation tests and essential medicines for the management of diabetes [4]. This translates into high rates of vascular complications which occurs early in the course of the disease [5], and which carries a high morbidity and mortality. Thus, after 6 years of diagnosis of diabetes in low-income settings, about 40% of patients with type 2 diabetes have diabetic retinopathy, of whom 15–17% have sight threatening retinopathy [5, 6]. Prevention of diabetic retinopathy and diabetes related blindness requires strict control of risk factors, regular eye checks with timely laser therapy [7]. Most patients with diabetes in low-income settings are first cared for by primary care physicians. There is evidence of a gap in the diagnosis and management of diabetes in low-income settings [2]. However, evidence on the standard of care to prevent diabetes related blindness, as well as the determinants of standard care are lacking in low-income settings. We report on the prevalence and determinants of comprehensive eye care in a group of patients with diabetes in a sub-Saharan African (SSA) setting.

## Main text

### Study design and setting

This was a cross-sectional study in the eye department of the Douala General Hospital between August and September 2006. It is a tertiary centre in the economic capital of Cameroon (a low-income setting located in sub-Saharan Africa), with a catchment population of over three million inhabitants. The eye department of this hospital served as the reference centre for entire Country and the sub region in terms of retinal pathologies, and likely to receive patients from all walks of life.

Participants were adult patients aged ≥ 18 years, of both sex having diabetes (type 1 or 2), who gave their inform consent. Pregnant women were excluded.

### Measurements

Before the comprehensive eye examination, each patient was interviewed using a standard questionnaire. The questionnaire used in this study was designed specifically for this study and was not pre-tested. Information registered are presented in Additional file 1. Patients then underwent a comprehensive eye examination. Outcome: The main outcome was a first ever comprehensive eye examination or at least a dilated fundus examination. The secondary outcome was haven been counseled on the risk of visual impairment and blindness due to diabetes. Possible determinants of having an eye examination were age, sex, residence, duration of diabetes, health insurance, level of education, sector of activity, treating physician, counseled on diabetes complications, associated hypertension, difficulties to reach the eye clinic, low visual acuity,

### Sample size and power

With an estimated catchment population of three million, an expected prevalence of diabetes to be 5.4 and 80% power, and an accepted error of 5%, the estimated number of participants needed for the descriptive study was 78.

### Statistical analysis

Data were analyzed using Epi-Info version 7. Baseline characteristics are presented by sex. Continuous variables are presented as mean ± standard deviation (SD), and discrete variables as frequencies and percentages, with their 95% confidence intervals. To calculate potential determinants (unadjusted Odds) for the first ever comprehensive eye examination, all variables were categorized. Chi squared test or Fisher exact test was used where appropriated to test for statistical significance. A two-sided P < 0.05 was considered statistically significant.

### Results

A total of 52 (67% of expected) patients were included in the study, of whom 31 (59.6%) were males. Their mean age was 55.9 ± 10.9 years, and ranged from 20 to 84 years. Baseline characteristics are summarized in Table 1. Most of the patients had type 2 diabetes (92.3%) that has been evolving for about 10 years. Most patients had secondary school level of education (38.5%) and lived in Douala (69.2%). Only nine (17.3%) had health insurance. The treating physician who referred the patient for eye examination was a diabetologist in 53.9% of cases. Most of the treating physicians also lived in Douala (73.1%).

**Table 1:** Baseline characteristics of participants

	Overall (N = 52)	Male (n = 31)	Female (n = 21)	P value
Mean age (years)	55.9 ± 10.9	54 ± 9.8	58.7 ± 12	0.128
Type 2 diabetes, %	92.3	96.8	85.7	0.144
Mean duration of diabetes (years)	9.5 ± 7.7	10.6 ± 8.6	7.8 ± 5.8	0.199
Level of education, %				
None	7.7	3.2	14.3	0.144
Primary	30.8	25.8	38.1	0.350
Secondary	38.5	41.9	33.3	0.536
University	23.1	29	14.3	0.221
Health insurance, yes, %	17.3	19.4	14.3	0.637
Sector of activity, %				
Primary	44.2	22.6	76.2	< 0.001*
Secondary	11.6	19.4	0	0.034*
Tertiary	44.2	58	23.8	0.016*
Treating physician, %				
Diabetologist	53.9	45.2	66.7	0.131
General practitioner	28.9	41.9	9.5	0.012*
Others	17.3	12.9	23.8	0.313
Reference to eye clinic, %				
Treating physician	53.9	41.9	71.2	0.040
Ophthalmologist	23.1	35.5	4.8	0.011*
Advised to consult	9.6	12.9	4.8	0.336
Self-consultation	13.5	9.7	19.1	0.335
Eye care counselling (yes), %	51.9	48.4	57.1	0.542
Dilated fundoscopy (yes), %	61.5	67.7	52.4	0.271
Number of fundoscopy, %				
None	38.5	32.3	47.6	0.271
One	19.2	22.6	14.3	0.461
Two	15.4	16.1	14.3	0.861
More than two	26.9	29	23.8	0.681
Referral time to actual consultation (weeks), %				
< 2	80.8	87.1	71.4	0.163
> 2	11.5	9.7	14.3	0.614
Unknown	7.7	3.2	14.3	0.144
Reaction to risk of blindness, %				
Worried	36.5	38.7	33.3	0.694
Indifferent	48.1	48.4	47.6	0.995
Can’t tell	15.4	12.9	19.1	0.547
Low visual acuity (VA < 3/10), %	42.3	41.9	42.9	0.944

Data are mean ± standard deviation, level of significance set at p < 0.05

* Significant difference

**Fig. 1:**
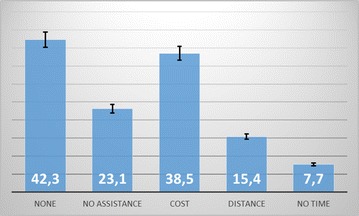
Difficulties faced while trying to afford a comprehensive eye examination

The main outcome is summarized in Table 1. About half of the patients (51.9%) have had counselling on the risk of visual impairment and blindness due to diabetes, and 32 (61.5%) have had a comprehensive eye examination. Of those with a first ever fundoscopy, 7 (21.9%) had the test performed within 1 year, and 25 (78.1%) had the test performed after 1 year of diagnosis. Thus, after an average of 10 years of the diagnosis of diabetes, 13.5% (7/52) of patients have had a comprehensive eye examination within 1 year of diagnosis. All fundoscopy was performed by an ophthalmologist. The possible determinants of a comprehensive eye examination are summarized in Table 2. Only dose with diabetes duration of more than 10 years were 7–24 times more likely to have a comprehensive eye examination. Those who admitted having no problem to seek comprehensive eye care were less likely to have a fundoscopy done. The main difficulties faced by patients in seeking eye care are summarized in Fig. 1. This is mostly due to the cost of healthcare, transportation, feeding and lodging. This was followed by lack of physical assistance (often a relative) to the Hospital.

**Table 2:** Determinants of a comprehensive eye examination

	Unadjusted odds ratio	95% confidence interval	P value
Age (years)			
≤ 50	1		
50–60	2	0.47–8.49	0.467
> 60	0.59	0.14–2.42	0.502
Sex			
Female	1		
Male	1.91	0.61–5.97	0.264
Duration of diabetes (years)			
0–5	1		
5–10	2	0.45–8.9	0.458
10–15	7	1.1–44.6	0.046*
> 15	24	2.5–230.7	0.004*
Diabetologist physician			
No	1		
Yes	0.67	0.23–2.07	0.250
Counselled on risk of blindness			
No	1		
Yes	1.13	0.37–3.5	0.526
Concerned about blindness			
No	1		
Yes	1.6	0.49–5.2	0.558
Health insured			
No	1		
Yes	2.52	0.47–13.58	0.239
Level of education			
None	1		
Primary	1.29	0.14–11.5	1.00
Secondary	2.33	0.26–20.7	0.578
University	1.4	0.08–13.6	1.00
Douala resident			
No	1		
Yes	1.38	0.41–4.57	0.412
Sector of activity			
Primary	1		
Secondary	3.85	0.39–38.6	0.362
Tertiary	1.19	0.37–3.9	1.00
Low visual acuity (VA < 3/10)			
No	1		
Yes	2.33	0.71–7.6	0.279
Hypertension			
No	1		
Yes	1.11	0.35–3.5	1.00
Difficulties to consult at eye clinic			
Yes	1		
No	0.3	0.09–0.97	0.050

Data are mean ± standard deviation, level of significance set at P < 0.05

* Significantly modified the risk of having comprehensive eye care examination

### Discussion

This study aimed to determine the prevalence and determinants of a comprehensive eye examination in a group of patients with diabetes in Cameroon. About 60% of the patients have had a comprehensive eye examination, and only about a fifth of these had an eye examination within the first year of diagnosis of diabetes. The duration of diabetes (more than 10 years) was associated with a 7–24 times more likely to have a comprehensive eye examination.

Most of the patients who presented for screening and/or treatment for sight threatening retinopathy were seen by internists/diabetologists. Similar findings of the likelihood of referral by internists were reported by several studies [8, 9]. Few general practitioners (who make up the bulk of the primary care physicians) refer patients with diabetes for eye examination. Similar findings in several studies showed that a significant number of primary care physicians do not follow the recommended guidelines set forth for diabetic eye care [9–13]. The findings suggest that general practitioners in this low-income setting lack awareness on the natural history of diabetic retinopathy, and of the success of current treatment. A similar finding was reported by Edwards [12].

The rate of awareness of the ocular complications of diabetes is low in this group of patients (51.92%) compared to that reported by Tapp et al. [14] in Australia, who found that 90% of participants were aware that diabetes was associated with visual impairment and blindness. This could be due to the implementation of education and awareness programs for diabetic retinopathy, and developing the role of primary care providers in screening for retinopathy in Australia [15]. This suggests that existing education and awareness strategies be reinforced with primary care providers occupying key role in our milieu.

A high proportion of patients (78.13%) had their first dilated fundus examination > 2 years after the diagnosis of diabetes, a rate far higher than that reported by Tapp et al. [14], who found 23%. We recommend the education of non-ophthalmologist to detect and to appropriately refer patients who are at risk for vision loss, as suggested by Awh et al. [16].

Health insurance status was not related to the patients’ ability to afford for quality health care.

In summary, patients with diabetes in this low-income setting in SSA do not receive a comprehensive eye care as recommended. Most patients will get an eye examination at least 10 years after the diagnosis of diabetes. The cause of this sub-optimal care is probably multifactorial, from lack of awareness on the part of the primary care physicians, to high cost of healthcare and associated ill-health on the part of the patients. Findings of this study revealed that most of diabetes patients have an important delay in eye examination. Considering the prevalence of this disorder in our context and importance of eye examination in detecting and diagnosis diabetes eye complications, such delay is worrying and must addressed. This will first required more studies with greater sample size which can investigate both determinants and outcomes of comprehensive eye examinations in order to find if there is a relation between this delay in eye examination and diabetes eye complications in these patients even. Considering the fact that diabetes eye examination can progress insidiously and given that eye examination is the only method to detect or diagnose such condition, it seems obvious that this delay in eye examination may influence development of eye complication but this need to be assessed by further studies. Also, measures must be taken to increase awareness of general population, diabetes individuals and general practitioners on the importance of having a comprehensive eye examination as soon as diagnosis is made or at least within 1 year as recommend.

## Limitations

Our findings should be interpreted in the light of the limitations. The sample size was small (less than 80% of expected), thus underpowered to detect statistically significant risk for not having a comprehensive eye examination. Also, this study was a specialist hospital based, and does not represent the general population of patients with diabetes. Thus, the proportion of those with eye examination reported could be overestimates. Despite these shortcomings, we provide baseline data for future large scale and community research. Also, our data was derived from patients so as to reduce reporting bias with the physician approach. However, there is a high risk of recall bias with this approach.

## Additional file

**Additional file 1.** Questionnaire.

## Abbreviations

SSA: sub Saharan Africa
WHO: World Health Organization
