# Supplementary material for: Prevalence and determinants of comprehensive eye care in a group of patients with diabetes: a cross-sectional study in a sub-Saharan African setting
Source: BMC Res Notes. 2018 Feb 27;11:157. doi: 10.1186/s13104-018-3265-1 (PMC5830332; doi:10.1186/s13104-018-3265-1)
Supplement: Supplementary file 1 — Additional file 1. Questionnaire. [file 13104_2018_3265_MOESM1_ESM.doc]

QUESTIONNAIRE

N°_______________Code_________________Date______________Phase______________

Contact tel_______________

I- WHO ARE THESE PATIENTS ?

1-Identification

A) Age________

B) Sex: 1=male; 2=female

C) Nationality

1=Cameroonian

2=Foreigner

D) Province of origine

1=EN, 2=NO, 3=AD, 4=ES, 5=SU, 6=CE, 7=LT, 8=OU,

9=NW, 10=SW, 11=None

e)Residence

1=Douala

2=Yaounde

3=Elsewhere

f) Occupation

1=Primary Sector

2=Secondary Sector

3=Tertiary Sector

2-Past medical history

- Diabete

A) Age at diagnosis (onset): years

1= <10, 2=10-20, 3=20-30, 4= >30, 5=Do not know

B) Duration of Diabetes (years)

1= <5, 2= 5-10, 3= 10-20, 4= 20-25, 5= >25

c) Type of Diabetes

1= type1, 2= type2, 3= other, 4= Don’t know

D) Treatment

1= Oral Hypoglycemic, 2= insulin, 3= diet only

4= Both insulin and Oral hypoglycemic

E) Last glycaemia (g/dl)

1= < 0,6; 2= 0,6-1,1; 3= 1,1-1,26; 4= >1,26

5= Do not know

- Associated risk factors

A) Hypertension: 1= No; 2= Yes

If Yes (2): Treatment: 1= No; 2= Yes

B) Tobacco use: 1=No; 2= Yes

C) Alcohol consumption > 26g/day

1= No; 2= Yes

D) Dyslipidemia: 1= No; 2= Yes

E) Anemia: 1= No, 2= Yes

F) Kidney disease: 1= No; 2= Yes

G) Heart disease: 1= No; 2= Yes

II/ AT WHAT STAGE OF THE DISEASE DO THEY PRESENT ?

1. Blood pressure(mmhg)

- Systolic: 1= <140; 2= 140-160; 3= >160
- Diastolic: 1= <80, 2= 80-90, 3= 90-100, 4= >100

B) Visual acuity

1= 3/10 – 10/10

2= 1/10 – 3/10 Right eye

3= 1/20 – 1/10

4= 1/50 – 1/20 Left eye

5= < 1/50

C) Tonometry (mmHg)

1= 10-20 Right eye

2= 20-30

3= >30 Left eye

D) Fundoscopy

1= No apparent Retinopathy Right eye

2= Non-proliferative DR

3= Proliferative DR Left eye

If 2 or 3(severity) ; 1= mild

2= moderate

3= severe

E) Maculopathy: 1= No; 2= Yes

If 2(severity); 1= mild

2= moderate

3= severe

1. complications

1= None

2= Vitreous haemorrhage

3= Retinal detachment

4=Neovascular glaucoma

5=cataract

1. Retinal angiography: 1=No; 2=Yes

-if 2, stage of disease (DR) 1=Mild

2=Moderate

3=Severe

1. Treatment protocol

1= Focal photocoagulation

2=panretinal photocoagulation

3=None (No treatment)

1. Post-intervention Visual acuity

1= 3/10 – 10/10

2= 1/10 - 3/10 Right eye

3= 1/20 - 1/10

4= 1/50 – 1/20 Left eye

5= < 1/50

j) Control angiography: 1= No; 2= yes

If 1= No; Why?................................................................................................................

K) Evolution

1= No change

2= Regression

3= Worsening

III WHAT ARE THE FACTORS THAT INFLUENCE EYE CARE SERVICES PROVIDED?

- 1. Health care giver factors

1. Is your doctor (who treats you diabetes)

1= Specialist in diabetes?

2= General practitioner?

3= Others?

ii) Where does he practice?

1=Douala

2=Yaounde

3= Others

iii) Has your doctor ever talked to you about the dangers

on your eyes as a diabetic patient? 1=No; 2=Yes; 3= Do not know

iv) Has drops ever been put into our eyes

and the back of your eyes examined?

1=No; 2=Yes

If yes (2), by – who? 1= Treating doctor

2=ophtalmologist

3=optometist

4=ophtalmic nurse

5= Do not Know

- - - At what time after onset of diabetes?

1= <1 year

2= >1 year

3= Donot know

- - - How did you come to know the eye clinic of the general hospital?

1= 1

2= 2

3= >2

4= Donot know

v) How did you come to know the eye clinic of the general hospital?

1= Referred by my doctor

2=Referred by my ophtalmologist

3=Advice from another person

4=I came on my own (personal decision)

B- PATIENT FACTORS

i)Is you health : 1= Insure d(prepaid)?

2= Non-Insured (fee-for-service)

ii) When told about the risk on your eyes, how did you feel?

1= Very worried and want something done

2= Normal, it is like other diseases or doesnot diturb now.

3= Others...................................................................................

iii) What is your level of education

1= None

2= Primary

3=Secondary

4= Universty

iv) What difficulties did you face coming here for eye care?

1=Inabilyty to pay for medical care

2= Lack of time

3= Lack no one to acompany here

4= Lack of money to cover cost of food,transport and lodging expenses

5= Others..............................................................................................

1. How long did it take you to come to this eye clinic after having after having been sent here?

1= < 2 weeks

2= 2 weeks – 1 month

3= 1- 2 months

4= > 2 months

if 3 and 4 , why?..................................................................................................................

..............................................................................................................

vi) How do you grade the treatment (photocoagulation)?

1= Satisfactory

2=Non-Satisfactory

3=cannot tell

if 2, why?...........................................................................................................................

..........................................................................................................................

Thank you
